# Comparisons against baseline within randomised groups are often used and can be highly misleading

**DOI:** 10.1186/1745-6215-12-264

**Published:** 2011-12-22

**Authors:** J Martin Bland, Douglas G Altman

**Affiliations:** 1 University of York, Heslington, York, YO10 5DD, UK; 2 Centre for Statistics in Medicine, University of Oxford ,Wolfson College Annexe, Linton, Road Oxford OX2 6UD, UK

**Keywords:** Baseline, significance, comparison, within-group, type I error, alpha, ageing

## Abstract

**Background:**

In randomised trials, rather than comparing randomised groups directly some researchers carry out a significance test comparing a baseline with a final measurement separately in each group.

**Methods:**

We give several examples where this has been done. We use simulation to demonstrate that the procedure is invalid and also show this algebraically.

**Results:**

This approach is biased and invalid, producing conclusions which are, potentially, highly misleading. The actual alpha level of this procedure can be as high as 0.50 for two groups and 0.75 for three.

**Conclusions:**

Randomised groups should be compared directly by two-sample methods and separate tests against baseline are highly misleading.

## Background

When we randomise trial participants into two or more groups, we do this so that they will be comparable in every respect except the intervention which they then receive. The essence of a randomised trial is to compare the outcomes of groups of individuals that start off the same. We expect to see an estimate of the difference (the "treatment effect") with a confidence interval and, often, a P value. However, rather than comparing the randomised groups directly, researchers sometimes look within groups at the change between the outcome measure from pre-intervention baseline to the final measurement at the end of the trial. They then perform a test of the null hypothesis that the mean difference is zero, separately in each randomised group. They may then report that in one group this difference is significant but not in the other and conclude that this is evidence that the groups, and hence the treatments, are different.

For example, a recent trial received wide media publicity as the first "anti-ageing" cream "proven" to work in a randomised controlled clinical trial [1]. Participants were randomised into two groups, to receive either the "anti-ageing" product or the vehicle as a placebo. Among other measures, the authors report the appearance of fine lines and wrinkles, measured on a scale of 0 to 8, at baseline and after six months.

The authors gave the results of significance tests comparing the score with baseline for each group separately, reporting the active treatment group to have a significant difference (P = 0.013) and the vehicle group not (P = 0.11). This was interpreted as the cosmetic "anti-ageing" product resulted in significant clinical improvement in facial wrinkles. But we cannot draw this conclusion, because the lack of a significant difference in the vehicle group does not mean that subjects given this treatment do not improve, nor that they do not improve as well as those given the "anti-aging" product. It is the sizes of the differences which is important; they should be compared directly in a two sample test.

The paper includes some data for the improvement in each group, 43% for the active group and 22% for controls. This was what was picked up by the media. No P value is given, but in the discussion the authors acknowledge that this difference was not significant. No confidence interval is given, either, the accepted preferred way to present the results of a randomised trial [2,3].

The *British Journal of Dermatology *published a letter critical of many aspects of this trial [4]. A different version subsequently appeared in *Significance *[5]. This happened, of course, only because the publicity generated by Boots brought the paper to the attention of JMB. The "anti-ageing" skin cream trial made us think again about this method of analysis, which we have written about several times before [6-10]. In this paper we try to present a clearer explanation for why within group analysis is wrong. It is a greatly expanded version of ideas we introducing briefly in our Statistics Notes series in the *British Medical Journal *[11].

### Simulation studies

We shall examine the statistical properties of testing within separate groups with a simulation. We consider the case where there is no true difference between the two treatments. Table 1 shows simulated data from a randomised trial, with two groups (A and B) of size 30 drawn from the same population, so that there is no systematic baseline difference between the groups. There is a baseline measurement, with standard deviation 2.0, and a final measurement, equal to the baseline plus a random variable with mean 0.0, standard deviation 1.0, plus a systematic increase of 0.5, half a standard deviation, in both groups.

**Table 1:** Simulated data from a randomised trial comparing two groups of 30, with no real difference.

**Group A**				**Group B**			
	
**Baseline**	**Final**	**Baseline**	**Final**	**Baseline**	**Final**	**Baseline**	**Final**
			
2.6	2.7	9.7	11.7	6.0	5.9	11.0	11.2
			
5.5	5.0	9.8	9.6	7.3	6.8	11.1	12.3
			
6.5	6.1	10.3	10.3	7.6	6.2	11.2	11.1
			
7.5	8.7	10.6	10.4	7.6	7.8	11.2	12.0
			
7.9	9.3	11.0	11.0	8.2	7.8	11.4	11.2
			
8.0	9.4	11.2	10.8	8.7	9.5	11.5	10.9
			
8.2	9.1	11.3	11.5	8.9	9.9	11.8	11.2
			
8.3	7.9	11.5	11.6	9.0	10.6	11.8	13.8
			
8.4	8.1	11.6	11.8	9.0	11.7	11.9	12.6
			
8.6	9.2	12.0	12.1	9.4	9.8	11.9	13.2
			
8.7	9.7	12.4	13.4	9.8	9.9	12.3	10.3
			
8.7	10.4	12.4	15.8	10.5	10.2	12.5	11.3
			
9.0	7.4	12.5	13.8	10.5	11.2	12.8	13.1
			
9.2	9.7	12.7	12.5	10.9	11.5	13.0	14.4
			
9.4	9.4	14.9	16.5	10.9	12.5	13.2	13.3

In this simulation, the proportion of possible samples which would give a significant difference between groups is 0.05. When the null hypothesis is true, this should be equal to the chosen type I error (alpha), which we have taken to be the conventional 0.05. There is no real difference, so the probability of a significant difference is 0.05, by definition. Within each group, there is an expected difference, so we can calculate the power to detect it, the probability of a significant difference, using the usual formula for a paired t test [12]. For the chosen difference of half a standard deviation of the differences, using significance level 0.05, the power is 0.75.

The usual way to analyse such data is to compare the mean final measurement between the groups using the two sample t method, or, better, to adjust the difference for the baseline measure using analysis of covariance or multiple regression [13]. For these data, using the two sample t method, we get difference between groups in mean final measurement (A - B) = -0.61, P = 0.3, and adjusting the difference for the baseline measure using regression we get difference = 0.19, P = 0.5. In each case the difference is not statistically significant, which is not surprising because we know that the null hypothesis is true: there is no true difference in the population.

There are other analyses which we could carry out on the data. For each group, we can compare baseline with final measurement using a paired t test. For group A, the mean increase is 0.48, which is statistically significant, P = 0.01; for group B the mean increase = 0.34, which is not significant, P = 0.08. The results of these significance tests are quite similar to those of the "anti-ageing" cream trial. We know that these data were simulated with an average increase of 0.5 from baseline to final measurement, so a significant difference in one group is not surprising. There are only 30 in a group so the power to detect the difference is not great. Indeed, only 75% of samples are expected to produce a significant difference, so the non-significant difference is not surprising, either.

We would not wish to draw any conclusions from one simulation. We repeated it 10,000 times. In 10,000 runs, the difference between groups had P < 0.05 in the analysis of covariance 458 times, or for 4.6% of samples, very close to the 5% we expect. For the 20,000 comparisons between baseline and final measurement, 15,058 had P < 0.05, 75.3%, corresponding to the 75% power noted above. Of the 10,000 pairs of t tests for groups A and B, 617 pairs had neither test significant, 5,675 had both tests significant, and 3,708 had one test significant but not the other. So in this simulation, where is no difference whatsoever between the two "treatments", 37.1% of runs produced a significant difference in one group but not the other. Hence we cannot interpret a significant difference in one group but not the other as a significant difference between the groups.

### How many pairs of tests would be expected to have a significant difference in one group and a non-significant difference in the other?

How many pairs of tests will have one significant and one non-significant difference depends on the power of the paired tests. First, we shall assume that there is no true difference between interventions and that the power of each test within the group is the same. This will be the case for equal-sized groups. If the population difference from baseline to final measurement is very large, nearly all within-group tests will be significant, whereas if the population difference is small nearly all tests will be not significant; in each case there will be few samples with only one significant difference. Intuitively the probability of one of the two tests being significant will rise in between these two extreme cases.

Looking at the problem mathematically, if there is no difference between groups and power of the paired t test to detect the difference between baseline and final measurement is *P*, the probability that the first group will have a significant paired test is *P*, the probability that the second will be not significant is 1 - *P *and the probability that both will happen is thus *P *× (1 - *P*). Similarly, the probability that the first will be not significant and second significant will also be (1 - *P*)×*P*, so the probability that one difference will be significant and the other not will be the sum of these probabilities, or 2*P *× (1 - *P*). It will not be 0.05, which it should be for a valid test between the groups.

When the difference in the population between baseline and final measurement is zero, the probability that a group will have a significant difference is 0.05, because the null hypothesis is true. The probability that one group will have a significant difference and the other will not is then 2*P *× (1 - *P*) = 2 × 0.05 × (1 - 0.05) = 0.095, not 0.05. So we expect 9.5% of samples to have one and only one significant difference. We ran 10,000 simulations of this completely null situation. In 10,000 runs, the difference between groups had P < 0.05 in the analysis of covariance 485 times, or for 4.9% of samples, very close to the 5% we expect. For the 20,000 comparisons between baseline and final measurement, 1,016 had P < 0.05, 5.1%, again very close to the 5% we expect. Of the 10,000 pairs of t tests for groups A and B, 9,008 pairs had neither test significant, 24 had both tests significant, and 968 had one test significant but not the other, 9.7%, very close to the 9.5% predicted by the theory but not to the 5% which we would want if this procedure were valid.

If the power of the within-group tests is 50%, as it would be here if the underlying difference were 37% of the within-group standard deviation, rather than 50%, as in our first simulation, then 2*P *× (1 - *P*) = 2 × 0.50 × (1 - 0.50) = 0.50. So we would expect 50% of two-sample trials to have one and only one significant difference. We ran 10,000 simulations of this situation, where the power for a within group difference is 50% but there is no between group difference. In 10,000 runs, the difference between groups had P < 0.05 in the analysis of covariance 490 times, or for 4.9% of samples. For the 20,000 comparisons between baseline and final measurement, 9,938 had P < 0.05, 49.7%, very close to the 50% power within the group which this simulation was designed to have. Of the 10,000 pairs of t tests for groups A and B, 2,518 pairs had neither test significant, 2,456 had both tests significant, and 5,026 had one test significant but not the other, 50.3%, very close to the 50% predicted by the theory but not to the 5% which we would want if this procedure were valid.

**Figure 1:**
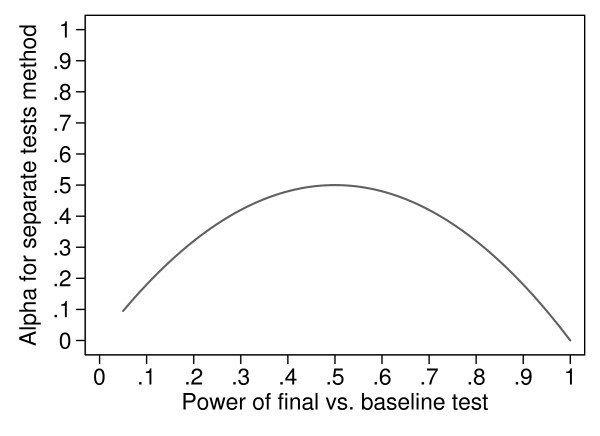
**Actual Type I error (alpha) for separate tests against baseline for two randomised groups against the power of the individual test of change from baseline**.

Figure 1 shows the actual alpha for a two-group trial against the power of the within-group test. This curve starts at *P *= 0.05, because this is the minimum possible power for a test with alpha = 0.05. The peak value is at *P *= 0.5 (the case just considered) and then actual alpha declines as *P *increases, because with high power both within-group tests are likely to be significant.

If the randomised groups represent populations which really are different after treatment, so that the null hypothesis of trial is not true, the calculations are more complicated. The power of the within-group tests will be different for the two groups. This is because the population difference will not be the same. If the power of the within-group test for group A is *P*_1 _and for group B it is *P*_2_, then the actual alpha for the within-groups procedure is *P*_1_×(1 - *P*_2_) + (1 - *P*_1_) ×*P*_2_. This will have its maximum, not surprisingly, when one test has high power and the other has low power. This might be the case when one treatment is ineffective, such as a placebo, though not when both treatments are active.

### Other examples of testing within randomised groups

The anti-ageing cream trial is by no means unusual in having tested within groups when the difference between groups is not significant. Altman [6] gave the following example. Toulon and colleagues divided patients with chronic renal failure undergoing dialysis into two groups with low or with normal plasma heparin cofactor II (HCII) [14]. Five months later, the acute effects of haemodialysis were examined by comparing the ratio of HCII to protein in plasma before and after dialysis. The data were analysed by separate paired Wilcoxon tests in each group.

Toulon and colleagues [14] published the data, which appear in Table 2, taken from Altman [6]. They analysed the data using two paired Wilcoxon tests. For the Low HCII group the before to after change was significant, P < 0.01. For the normal HCII group the difference was not significant, P > 0.05.

**Table 2:** HCII/protein ratio in two groups of patients (14 *et al. *1987, reported by Altman 1991)

Group 1 (low HCII)		Group 2 (normal HCII)	
**Before**	**After**	**Before**	**After**

1.41	1.47	2.11	2.15

1.37	1.45	1.85	2.11

1.33	1.50	1.82	1.93

1.13	1.25	1.75	1.83

1.09	1.01	1.54	1.90

1.03	1.14	1.52	1.56

0.89	0.98	1.49	1.44

0.86	0.89	1.44	1.43

0.75	0.95	1.38	1.28

0.75	0.83	1.30	1.30

0.70	0.75	1.20	1.21

0.69	0.71	1.19	1.30

What should they have done? They could have done a two sample t test between groups on the ratio before dialysis minus ratio after. This gives *t *= 0.16, 22 d.f., P = 0.88, or for the log transformed data *t* = 1.20, P = 0.24. The variability is not the same in the two groups, so they might have done a two sample rank-based test, the Mann Whitney U test. This gives *z *= 0.89, P = 0.37. So either way, the difference is not statistically significant.

In that example, we could tell what the between groups test would give because the raw data were given. We cannot usually tell what the between group comparison would show when researchers test within groups. Bland and Peacock gave the next two examples [9]. In a randomized trial of morphine vs. placebo for the anaesthesia of mechanically ventilated pre-term babies, it was reported that morphine-treated babies showed a significant reduction in adrenaline concentrations during the first 24 hours (median change -0.4 nmol/L, P < 0.001), which was not seen in the placebo group (median change 0.2 nmol/L, P < 0.79 (sic)) [15]. There is no way to test whether the between group difference is significant. Even though the median changes in this example are in opposite directions, this does not imply that there is good evidence that the treatments are different.

**Figure 2:**
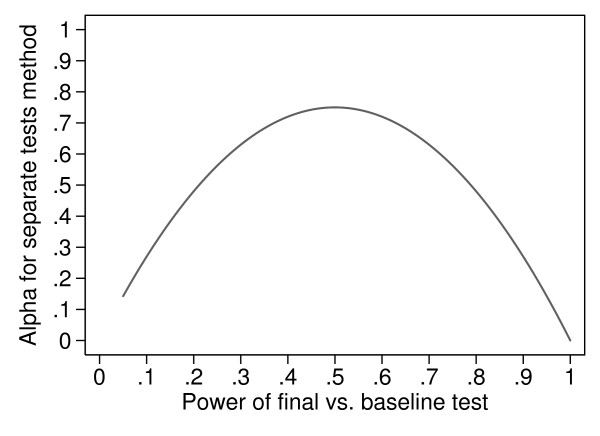
**Actual Type I error (alpha) for separate tests against baseline for three randomised groups against the power of the individual test of change from baseline**.

In a study of treatments for menorrhagia during menstruation, 76 women were randomized to one of three drugs [16]. The effects of the drugs were measured within the subjects by comparing three control menstrual cycles and three treatment menstrual cycles in each woman. The women were given no treatment during the control cycles. For each woman the control cycles were the three cycles preceding the treatment cycles. The authors reported that patients treated with ethamsylate used the same number of sanitary towels as in the control cycles. A significant reduction in the number of sanitary towels used was found in patients treated with mefenamic acid (P < 0.05) and tranexamic acid (P < 0.01) comparing the control periods with the treatment periods. For three groups, when the differences between interventions are all zero, the probability that one test will be significant and the other two will not is 3*P*(1 - *P*)^2 ^and that two tests will be significant and one not significant is 3*P*^2^(1 - *P*). The probability of getting at least one significant and one not significant test between baseline and final measurement is, therefore, 3*P*(1 - *P*)^2 ^+ 3*P*^2^(1 - *P*), which is equal to 3*P*(1 - *P*). The graph of this probability is shown in Figure 2. The value when all the null hypotheses within groups are true is 0.14, even greater than for two groups, and the maximum value, when *P *= 0.5, is 0.75. So if we compare three groups with within-group tests, and the interventions all have identical effects, we could have an actual alpha for the test as high as 0.75, rather than the 0.05 we need for a valid test.

Sometimes authors test within groups when a between groups procedure would have given a significant difference. Kerrigan and colleagues [17] assessed the effects of different levels of information on anxiety in patients due to undergo surgery. They randomized patients to receive either simple or detailed information about the planned procedure and its risks. Anxiety was measured again after patients had been given the information. Kerrigan *et al. *calculated significance tests for the mean change in anxiety score for each group separately. In the group given detailed information the mean change in anxiety was not significant (P = 0.2), interpreted incorrectly as "no change". In the group given simple information the reduction in anxiety was significant (P = 0.01). They concluded that there was a difference between the two groups because the change was significant in one group but not in the other. As before, we should compare the two groups directly. We carried out an alternative analysis which tested the null hypothesis that, after adjustment for initial anxiety score, the mean anxiety scores are the same in patients given simple and detailed information. This showed a significantly higher mean score in the detailed information group [7].

A different reason for testing within groups was given by Grant and colleagues [18]. They compared acupuncture with Transcutaneous Electrical Nerve Stimulation (TENS) in patients aged 60 or over with a complaint of back pain of at least 6 months duration. Patients were randomly allocated to 4 weeks treatment with acupuncture or TENS. The intention was to compare the two treatments. The authors report that, if 75% of patients responded to acupuncture and 40% to TENS then a sample size of 30 in each group would give the trial a power of 80% to detect statistical significance at a probability level of P = 0.05.

Four outcome measures were recorded: (1) visual analogue scale (VAS); (2) pain subscale of the 38-item Nottingham Health Profile Part 1 (NHP); (3) number of analgesic tablets consumed in the previous week; (4) spinal flexion measured from C7 to S1.

The two groups appeared different at baseline, with patients in the acupuncture group having higher VAS and NHP pain scores, reduced spinal flexion and lower tablet consumption compared to the TENS group. The authors carried out significant tests comparing the randomised groups for these baseline variables. They reported that the differences were "of borderline statistical significance: P = 0.064 for NHP, P = 0.089 for VAS, P = 0.10 for tablets and P = 0.16 for flexion". We think that these tests are meaningless, because if the groups were allocated randomly we know the null hypothesis, which is about the population, not the sample, is true [19]. Grant and colleagues thought that these baseline differences would make post-treatment comparisons between groups difficult as even a small imbalance between initial values might affect the pain relief obtained by different treatments. They therefore analysed each group separately, comparing post-treatment final measurement with baseline. They obtained highly significant pain reductions in each group. They made some qualitative comparison between the treatments, but completely abandoned their original objective. They could have done this by using an analysis which adjusted for the baseline using regression. They should have done this whether or not the groups differed at baseline, because it would reduce variability and so increase power and precision.

Calculating a confidence interval for each group separately is essentially the same error as testing within each group separately. Bland [8] gave this example. Salvesen and colleagues [20] reported follow-up of two randomized controlled trials of routine ultrasonography screening during pregnancy. At ages 8 to 9 years, children of women who had taken part in these trials were followed up. A subgroup of children underwent specific tests for dyslexia. The test results classified 21 of the 309 screened children (7%, 95% confidence interval 3% to 10%) and 26 of the 294 controls (9%, 95% confidence interval 4% to 12%) as dyslexic. They should have calculated a confidence interval for the difference between prevalences (-6.3 to +2.2 percentage points) or their ratio (0.44 to 1.34), because we could then compare the groups directly.

Some authors test or estimate between groups, but then use a within groups test to suggest that, even though there is insufficient evidence for a difference between groups, the test against baseline suggests that their test treatment is superior. In a study of spin put on the results of 72 statistically non-significant randomised controlled trials, Boutron and colleagues [10] identified focus on a statistically significant within-group comparison as a common method to slant interpretation of results in favour of the test treatment in 11% (95% CI 5% to 21%) of abstracts and in 14% (7% to 24%) of results sections. In fairness, they note that all these articles also reported the statistically non-significant results for the primary outcome in the abstract and in the main text.

## Discussion

Using separate paired tests against baseline and interpreting only one being significant as indicating a difference between treatments is a frequent practice. It is conceptually wrong, statistically invalid, and consequently highly misleading. When the null hypothesis between groups is true, the Type I error can be as high as 50%, rather than the nominal 5%, and even higher when more than two groups are compared.

The actual alpha for the flawed separate tests method is a minimum when the null hypothesis comparing outcome with baseline is true, but this is not likely to be the case in practice. The condition of patients is likely either to improve or to deteriorate over time, depending on the natural history of the disease; people either get better or worse. Placebo effects or regression towards the mean may also lead to changes over time. Hence the population mean difference is likely to be non-zero and so the power to detect it will be greater than 0.05, and the actual alpha for two within-group tests will be greater than the 0.095 found when all null hypotheses are true. Only when the power within the group is very high, with either large differences from baseline or large sample sizes, will the actual alpha be lower than 0.095. Tests comparing the final measurement with baseline are useless in most cases. We cannot conclude that a treatment has effect because a before vs. after test is significant, because of natural changes over time and regression towards the mean [21]. We need a direct comparison with a randomised control.

We wondered whether this practice is declining with what are, we hope, improvements in medical statistical education and in research quality. A survey of 80 trial reports in major journals in 1987 found that in 8 (10%) trials analyses were done only within treatment groups [22]. A survey reported in 2011 of 161 trials identified from Cochrane reviews and published between 1966 and 2009 found that 16 (10%) reported a within-group comparison only [23]. There is not much evidence of progress.

This practice is widespread in non-randomised studies also. In a review of 513 behavioural, systems and cognitive neuroscience articles in five top-ranking journals, 79 articles were found to contain this incorrect procedure [24]. These authors reported that an additional analysis suggested that this method of analysis is even more common in cellular and molecular neuroscience. It is not just used as the main analysis for comparing randomised groups.

Why do researchers do this? We know of no statistics text books which advocate this approach and ours explicitly warn against it [6,8,9]. To anybody who understands what "not significant" means, it should be obvious that within-group testing is illogical. It should also appear so to anyone who has attended an introductory research methods course, which would have mentioned the importance and use of a control group. Do researchers invent this for themselves, or do they copy published papers which have gone down this misleading road? Every statistical advisor has come across consulters who say, when told that their proposed method is wrong, that some published paper has used it, so it must be correct. Simple ignorance could be the explanation and we have no way of knowing in any particular case how the mistake came about. We should not assume that an author testing within groups is doing it to hide an underlying non-significant difference and one of the examples given above showed a significant difference when a valid analysis was used. But as Darrell Huff wrote in 1954: "As long as the errors remain one-sided, it is not easy to attribute them to bungling or accident." [25].

## Conclusions

We think that randomised groups should be compared directly by two-sample methods and that separate tests against baseline are highly misleading. We also think that trialists should produce estimates with confidence intervals rather than significance tests [2,3].

## Competing interests

The authors declare that they have no competing interests.

## Authors' contributions

JMB carried out the simulations and the algebra. JMB and DGA each contributed examples and wrote and agreed the manuscript jointly.
